# Lessons from linking bio‐ and ecological traits to stoichiometric traits in stream macroinvertebrates

**DOI:** 10.1002/ece3.9605

**Published:** 2022-12-08

**Authors:** Miriam Beck, Elise Billoir, Vincent Felten, Albin Meyer, Philippe Usseglio‐Polatera, Michael Danger

**Affiliations:** ^1^ CNRS, LIEC Université de Lorraine Metz France; ^2^ LTER‐“Zone Atelier Moselle” Metz France; ^3^ Institut Universitaire de France (IUF) Paris France

**Keywords:** body stoichiometry, ecological stoichiometry, functional traits, global stressor, nitrogen, phosphorus

## Abstract

Ecologists rely on various functional traits when investigating the functioning of ecological systems and its responses to global changes. Changing nutrient levels, for example, can affect taxa expressing different trait combinations in various ways, e.g., favoring small, fast‐growing species under high phosphorus conditions. Stoichiometric traits, describing the elemental composition of organism body tissues, can help in understanding the mechanisms behind such functional shifts. So far, mainly life‐history traits have been related to body stoichiometry (e.g., the growth rate hypothesis) on a limited number of taxa, and there is little knowledge of the general link between stoichiometric and other functional traits on a taxonomically large scale. Here, we highlight this link in the freshwater macroinvertebrates, testing predictions from underlying trait‐based and Ecological Stoichiometry Theory (EST) in >200 taxa belonging to eight larger taxonomic groups. We applied a series of multivariate analyses on six of their stoichiometric traits (%C, %N, %P, C:N, C:P, and N:P) and 23 biological and ecological traits. We found significant relationships between stoichiometric traits and other types of traits when analyzing single‐trait and multi‐trait profiles. Patterns found within traits related to organism development or nutrient cycling were in line with our assumptions based on EST, e.g., traits describing predators were associated with high %N; traits suggesting a fast development (small maximum body size and high molting frequency) with high %P. Associations between ecological traits and body stoichiometry could be explained by the longitudinal stream gradient: Taxa preferring headwater habitats (i.e., high altitude, coarse substrate, and cold temperature) exhibited high %N and %P. Demonstrating the link between stoichiometric and both bio‐ and ecological traits on a large diversity of taxa underlines the potential of integrating stoichiometric traits into ecological analyses to improve our understanding of taxonomic and functional responses of communities—and ecosystems—to changing environmental conditions worldwide.

## Graphical abstract

Studying a large number of taxa (>200), we demonstrated a general link between stoichiometric traits (i.e., a taxon body elemental composition) and other biological or habitat‐related traits. The patterns we found were in line with concepts within the Ecological Stoichiometry framework, thus underlining the potential of using stoichiometric traits to understand (taxonomic and functional) community responses towards global nutrient stressors.

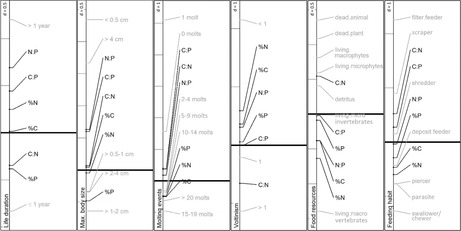

## 1 INTRODUCTION

Ecologists describe communities using two different ways: Taxonomic units as descriptors of assemblages or traits intended to be proxies of functions. While the first approach gives a better picture of phylogenetic signals, the latter one can provide a functional view relating species distributions to environmental factors up to the ecosystem scale (Cadotte et al., 2011; McGill et al., 2006). Functional traits encompass morphological, physiological, or phenological characteristics and ecological preferences (e.g., substrate, temperature) of an organism. They impact the fitness of this organism and influence its interactions with the environment and other organisms (Violle et al., 2007). Since taxa of distinct taxonomic lineages may express more similar traits than closer related ones (Pilière et al., 2016; Usseglio‐Polatera et al., 2000), taxonomic and functional diversity can provide distinct—sometimes divergent—but complementary information (Crabot et al., 2020, 2021). The trait structure of assemblages thereby is far less constrained by biogeography than their taxonomic structure because trait‐based functions persist within and among regions even when species pools are changing (Archaimbault et al., 2005; Charvet et al., 2000; Statzner et al., 2001). This enables addressing also large spatial scales. Since species have to find a trade‐off between their ecological demands and tolerances—closely related to key life‐history traits—and the abiotic/biotic features of habitats, community trait composition is closely depending on environmental parameters.

Changes in nutrient concentrations, the main stressor in many (aquatic) environments, seem to differently affect taxa differing in life‐history traits or feeding types (Cross et al., 2005, 2006; Evans‐White & Lamberti, 2009; Townsend et al., 2008). For example, while short‐lived chironomids increased their growth rate and production under high nutrient conditions, the longer‐lived *Tallaperla* sp. (Plecoptera) remained unaffected (Cross et al., 2005). Manipulative and observational studies have reported increasing relative abundances of small, short‐lived, fast‐growing, and ‐reproducing taxa under high nutrient conditions (Ortiz & Puig, 2007; Prater et al., 2015; Singer & Battin, 2007; Statzner & Bêche, 2010).

Including stoichiometric traits as a third set of metrics, besides traditional functional traits and taxonomic units, might help to understand the mechanism behind these observations. Stoichiometric traits describe an organism body nutrient content (e.g., P‐content, C:P ratio), which is assumed to reflect its demand for the corresponding nutrient. The framework of ecological stoichiometry (“Ecological Stoichiometry Theory,” EST) investigates the link between environmental nutrient concentrations and the nutrient content in organisms' body tissues in a given ecological context (Sterner & Elser, 2002). One well‐known example of a link between stoichiometric and functional traits is the growth rate hypothesis (GRH; Elser et al., 1996), which explains the increasing body P‐content with increasing growth rate by the increased demand for P‐rich rRNA necessary to achieve fast growth. Vice versa, slow‐growing taxa have a lower P‐demand, and thus a lower P‐content (Acharya et al., 2004; Elser et al., 2003). For other traits such as feeding type (Fagan et al., 2002; González et al., 2017), body size (Liess & Hillebrand, 2005; Meunier et al., 2016), or development rate (Beck et al., 2021), differences in stoichiometry between taxa expressing different trait adaptations have also been identified. Linking such growth‐related assumptions to nutrient concentrations in the environment, Singer and Battin (2007) and Prater et al. (2015) showed that the shift towards small, fast‐growing taxa under high nutrient conditions goes along with increasing abundances of P‐rich taxa. This indicates the role that stoichiometry could play in understanding the response to ecological stressors in community composition and functions.

Thus, large efforts have been made over the last decades to investigate the relationship between stoichiometric traits and functional traits and/or the environment, providing knowledge that has helped in understanding the mechanisms behind ecological processes from the individual to ecosystem level (Sardans et al., 2021). However, most works are limited to some laboratory model taxa or taxonomically reduced communities and traits were rarely addressed in combination. By extending the number of traits and taxa compared with previous studies and applying a multivariate approach, we therefore aim to investigate more precisely the general links between functional and stoichiometric traits. We focus on freshwater benthic macroinvertebrates, a group widely used for biomonitoring, representing high diversity (in terms of physiology, adaptations to environmental conditions, ecological value, and sensitivity to stressors) and for which detailed information on their traits is available (Sarremejane et al., 2020; Schmidt‐Kloiber & Hering, 2015; Tachet et al., 2010). Our study comprises >200 taxa of Turbellaria, Oligochaeta, Clitellata, Bivalvia, Gastropoda, Arachnida, Malacostraca, and Insecta. Besides biological traits referring to characteristics of the organism itself, we also included ecological traits that describe environmental preferences or tolerances of taxa.

Based on what is known from previous works and observations for specific taxa and/or conditions, we derived hypotheses about the general link between stoichiometric and functional traits (here distinguished as either biological or ecological traits), which we tested over a wide taxonomic range of macroinvertebrates (Table 1). Biological traits that are directly linked to nutrient cycling (feeding type, preferred food resources) or organism growth rate and life history (maximum size, voltinism, life duration, number of reproductive events, or number of molting events per individual) should exhibit the strongest associations with stoichiometric traits. The ecological traits should in turn be indirectly related to organism body stoichiometry. Substrate preferences for example are linked to resource preferences and therewith also to feeding mode. The association observed for substrate preferences should thus be in line with those found in the nutrient‐linked traits. We additionally expect variation in the patterns of stoichiometric/ecological trait relationships along the longitudinal gradient of rivers, as categories describing ecological traits should be expressed differently along the river continuum (e.g., temperature, flow velocity, substrate). Due to the linkages between some functional traits, we expect the stoichiometric responses of such traits to be linked to one another (e.g., feeding type and food resource, substrate and locomotion, feeding type and locomotion or substrate attachment). More precise hypotheses on the expected associations between stoichiometric traits and biological traits or ecological traits are provided in Table 1.

**TABLE 1:** Expected associations between functional and stoichiometric traits (a detailed description of the traits and all trait categories are provided in Table 2)

Functional trait	Hypotheses
Biological	
Development	
Maximum body size	Small maximum body sizes → fast growth rates and thus high body %P and low C:P and N:P (GRH, Carrillo et al., 2001; Elser et al., 1996; Main et al., 1997; Meunier et al., 2016)
Life duration, Molting events, Voltinism	Trait categories indicating fast development (short life duration, several generations per year, high frequency—leading also to high number—of molting events) → high body %P, low C:P and N:P due to fast growth (cf. “max. body size” above (Beck et al., 2022) and increased P‐demand during molts (Villar‐Argaiz et al., 2020)
Feeding	
Feeding habits, Food resources	Linked to nutritional quality of the resource: Nutrient‐rich (in)vertebrates, consumed by swallowers/chewers → high body %N and %P, low C:P and N:P; Living plant‐based resources (macroalgae, microphytes) of intermediate quality, consumed by filter‐feeders or scrapers → intermediate values of body %N and %P; Nutrient‐poor resources (detritus, dead plants or animals), consumed by deposit‐feeders and shredders → low body %N and %P, high C:P and N:P (Cross et al., 2003; Evans‐White et al., 2005; Lauridsen et al., 2012)
Others	
Locomotion	Linked to energy and muscle investment: Swimmer → high body %N due to investment in muscle tissue (González et al., 2018; Stjernholm & Karlsson, 2008) to perform swimming; Attached → low %N because low muscle demand for locomotion
Ecological	
Altitude, Longitudinal distribution	Linked to substrate and resource preferences: Alpine and upstream river sections with dominating leaf litter/detritus and boulders/flags (following RCC; Vannote et al., 1980) → high body C:P and N:P; Lowland and metapotamon/estuary with high quantities of mud/sand and macroalgae/microphytes (following RCC) and larger anthropogenic nutrient inputs (Aguilera et al., 2012; Paisley et al., 2011) → high body %N and %P due to nutrient‐richer resources (cf. “Feeding habits”)
Substrate	Linked to nutritional quality of the substrate (cf. “Feeding habits”): Detritus/litter → low body %N and %P, high C:P and N:P due to low nutritional quality; Macro‐/microphytes → higher body %N and %P due to higher nutritional quality
Temperature	Linked to metabolism: Higher energy demand (due to C loss from increased respiration) under warmer temperatures and preferences for carbon‐rich resources (Boersma et al., 2016; Hamburger & Dall, 1990) → low body C:N and C:P ratios Probably counteracting with: faster growth/development rates under warmer temperature (Hoegh‐Guldberg & Pearse, 1995; Levinton, 1983; Nilsson‐Örtman et al., 2012) → high %P (Janssens et al., 2015; cf. “development‐related traits”)
Trophic status	Eutrophic environments → high body %N and %P due to increased food quality of basal resources under high nutrient conditions (Cross et al., 2003; Danger et al., 2016; Demi et al., 2019)
Velocity	Linked to mobility (cf. “locomotion”): Fast → high body %N due to more active mobility, which requires N‐rich muscle tissue; Slow/null → low body %N due to lower proportion of muscles

## 2 MATERIAL AND METHODS

### 2.1 Datasets

Stoichiometric trait information was obtained from a database comprising 259 macroinvertebrate taxa and the elemental mass content of %C, %N, %P, and the corresponding molar ratios (C:N, C:P, N:P) as taxon mean values (Beck et al., 2022). It has been constructed mainly based on field samplings, supplemented by literature research. For this study, only taxa at the family level or finer (i.e., genus, species) were considered for Turbellaria, Clitellata, Bivalvia, Gastropoda, Malacostraca, and Insecta. Arachnida and Oligochaeta were considered as such.

Functional trait information was obtained from a database comprising information about >400 macroinvertebrate taxa and their biological and ecological traits (updated version of Tachet et al., 2010). The trait “number of reproductive cycles per individual” was taken from Beck et al. (2022) and “number of molting events” was coded for this study. The trait profiles were described via fuzzy coding: Each trait is represented by several trait categories, and for each category, the affinity of a taxon is given by a score ranging from 0 (= no affinity) to 5 (= high affinity) (Chevenet et al., 1994). The trait profiles of taxa thereby are taking into account the intrataxon variation and describe the variety of attributes potentially exhibited by a given taxon (e.g., by the successive aquatic instars during development). When only a very low proportion of rare taxa expressed a trait category, it was merged with the most biologically—or ecologically—similar category of the same trait, by summing their respective relative frequency values. As an example, the two extreme categories at both ends of the gradient of “maximum body size” (i.e., “<0.25 cm” and “>8 cm”) were merged with their nearest categories (i.e., “≥0.25–0.50 cm” and “≥4–8 cm,” respectively), into the new categories “≤0.5 cm” and “>4 cm.” All these modifications of trait categories are provided in the Supplementary Material (S1).

For the analysis, the relative frequencies of trait categories were calculated for each trait and each taxon, based on these affinity scores. In case of missing trait information due to differences in taxonomic resolution between the two databases, the trait information of the nearest coarser or finer taxonomic level (up to family level) was attributed when available (i.e., a value from a family or species to a genus but not from a species to a family except for obvious traits such as aquatic life stages in Mollusca). Thereby, traits known to be highly variable or showing large variation in their values among the finest taxonomic levels described in the database were not estimated (i.e., maximum body size, number of reproductive cycles).

For some functional traits, we do not list clear a priori hypotheses on associations to stoichiometric traits (Table 1) due to complex relationships between trait expressions (for example the selection of a trait category under certain environmental conditions can depend on the selection of categories of other traits). For other traits, we suspect an important role of phylogeny due to general differences among major invertebrate lineages (e.g., aquatic life stage, dispersal, resistance form, respiration). Nevertheless, we decided to include all these traits in the analyses as they are part of the organisms' trait profile and such an analysis can reveal some unexpected and potentially interesting relationships with stoichiometric traits. In total, we analyzed 13 biological and 10 ecological traits (Table 2). A list of taxa is provided in the Supplementary Material (S2). Insects were by far the largest group of taxa represented in this study, but in general, they make up the largest proportion of a macroinvertebrate community, at least in springs and the upper, wadeable reaches of streams and rivers (Usseglio‐Polatera & Beisel, 2002). Thus, our study list of taxa is fairly representative of the natural composition of macroinvertebrate assemblages in such systems.

**TABLE 2:** Functional traits and their corresponding trait categories

Type	Functional trait	Categories
Biological		
Development	Life duration	≤1 year, >1 year
	Maximum body size	≤0.5, >0.5–1, >1–2, >2–4, >4 cm
	Molting events	0, 1, 2–4, 5–9, 10–14, 15–19, >20 (= number of molting events per lifetime)
	Reproductive cycles	1, 2, >2–6, >6 (= number of reproductive cycles per lifetime)
	Voltinism	<1, 1, >1 (= number of generations per year)
Feeding	Feeding habits	Deposit‐feeder, filter‐feeder, piercer, parasite, scraper, shredder, swallower/chewer
	Food resources	Dead animal (≥1 mm), dead plant (≥1 mm), detritus (<1 mm), living macrophytes, living microphytes, living microinvertebrates, living (in)vertebrates
Others	Aquatic life stages	Egg, larva, nymph, adult
	Dispersal	Aquatic‐active, aquatic‐passive, aerial‐active, aerial‐passive
	Locomotion	Attached, burrower, crawler, interstitial, swimmer
	Reproduction mode	Ovoviviparity, isolated eggs (free), isolated eggs (cemented), clutches (cemented/fixed), clutches (free), clutches (in vegetation), clutches (terrestrial), asexual reproduction
	Resistance forms	Eggs/statoblasts, housing/cocoons, diapause/dormancy, none
	Respiration	Tegument, gill, plastron/spiracle
Ecological	Altitude	Lowland, piedmont, alpine
	Longitudinal distribution	Crenon, epirithron, metarithron, hyporithron, epipotamon, metapotamon, estuary, outside river
	Low pH tolerance	≤4, >4–4.5, >4.5–5, >5–5.5, >5.5–6, >6
	Salinity tolerance	Brackish water, freshwater
	Saprobity	Xenosaprobic, oligosaprobic, β‐mesosaprobic, α‐mesosaprobic, polysaprobic
	Substrate	Detritus/litter, flags/boulders/cobbles/pebbles, gravel, macrophytes, microphytes, mud, sand, silt, twigs/roots
	Temperature	Eurythermic, psychrophilic, thermophilic
	Transversal distribution	Banks/connected side‐arms, lakes, marshes/peat bogs, ponds/pools/disconnected side‐arms, river channel, temporary waters
	Trophic status	Eutrophic, mesotrophic, oligotrophic
	Velocity	Null, slow, medium, fast

### 2.2 Data analysis

The relationships between the functional trait profiles of taxa and their stoichiometric profiles were investigated via co‐inertia analysis (Dolédec & Chessel, 1994). This multivariate approach identifies the common structure in two tables, both describing the same set of individuals (taxa in this study). The co‐inertia analysis thereby follows a simple ordination analysis applied to each set of variables (stoichiometric and bio‐/ecological traits) separately. Since both tables are analyzed simultaneously as a symmetric canonical ordination technique, neither of them is treated as a table of explanatory or response variables. This is important since it is not possible to make statements about the direction of causation, i.e., whether a certain stoichiometric profile causes the expression of certain biological or ecological traits, or whether these traits cause the observed body stoichiometry. An RV coefficient was calculated as the sum of eigenvalues of the co‐inertia analysis (= total co‐inertia) divided by the square root of the product of the squared sum of eigenvalues (= total inertia) from the individual (i.e., one‐table) analyses:

$$\mathrm{RV}=\frac{\text{coinertia}\;\left(\mathrm{XY}\right)}{\sqrt{\text{inertia}\;\left(X\right)\times \text{inertia}\;\left(Y\right)}}$$

The RV value varies within the [0–1] range, with a high value indicating a high degree of co‐structure.

Co‐inertia analyses (CoI) were conducted (1) separately for each functional trait or (2) considering the “complete trait profile” taking into account simultaneously all the biological traits and ecological traits. The procedure was the same in both approaches. Prior to co‐inertia analysis, a Fuzzy Correspondence Analysis (FCA; Chevenet et al., 1994) was applied to the fuzzy‐coded functional trait profile array. Stoichiometric data were analyzed by normalized Principal Component Analysis (PCA). To correct for the characteristics of ratio data that can lead to biased results, the stoichiometric ratios (C:N, C:P, N:P) were log‐transformed before manipulation as advised by Isles (2020). Mass element stoichiometry was used without prior transformation. A co‐inertia analysis was applied to the results of each couple of unconstrained ordinations. The significance of RV‐values, i.e., of the co‐structures, was tested via permutation tests (9999 permutations). Since the stoichiometric trait information was used in all the separate analyses and in the analysis of the complete trait profiles, the p‐values were corrected for multiple testing with the Bonferroni method.

Due to the requirements of the statistical methods applied, only taxa with complete information (both bio‐/ecological and stoichiometric traits) were used in the analyses, leading to slightly varying lists of taxa used in the different analyses (*n* = 160–224 for the CoI analyses of single functional traits, *n* = 131 for the CoI analysis of the complete set of functional traits).

All analyses were performed using R software (R Development Core Team, 2019, version 3.5.2) and the package ade4 (Dray & Dufour, 2007).

## 3 RESULTS

### 3.1 Single traits

Seventeen significant co‐structures were observed between biological or ecological traits and stoichiometric traits, with RV‐coefficients ranging between 0.251 for “salinity tolerance” and 0.069 for “voltinism” (Table 3). For all the traits, the largest part of the co‐structure was projected on the first axis (66.15%–98.83% for traits with at least three trait categories, i.e., allowing for at least two axes). The following detailed description will thus focus on the position of trait categories along this axis, but when the second axis also captured a substantial part of the co‐structure (>15%), this will be discussed as well.

**TABLE 3:** Results of the co‐inertia analyses between individual functional traits and stoichiometric traits (%C, %N, %P, C:N, C:P, N:P) of macroinvertebrates

Type	Functional trait	RV	*n*_mod_	*n*_taxa_	Projected inertia (%)	
					Axis 1	Axis 2
*Biological*						
Development	Life duration	**0.0816**	2	224	100.00	NA
	Maximum body size	**0.0968**	5	160	88.98	10.70
	Molting events	**0.1606**	7	223	90.52	6.82
	Reproductive cycles^X^	0.0583	4	182	95.53	4.08
	Voltinism	**0.0694**	3	224	93.55	6.45
Feeding	Feeding habits	**0.1416**	7	224	70.81	26.66
	Food resources	**0.1508**	7	224	79.82	19.20
Others	Aquatic life stages^X^	**0.2504**	4	224	94.75	5.20
	Dispersal^X^	**0.1600**	4	224	85.20	14.45
	Locomotion	0.0198	5	224	66.90	29.89
	Reproduction mode^X^	**0.0980**	8	224	78.79	13.95
	Resistance forms^X^	0.0495	4	220	66.95	32.04
	Respiration^X^	0.0324	3	224	91.94	8.06
*Ecological*	Altitude	**0.0804**	3	224	98.83	1.17
	Longitudinal distribution	**0.1104**	8	224	66.15	32.26
	Low pH tolerance^X^	0.0234	6	220	84.50	14.85
	Salinity tolerance^X^	**0.2508**	2	224	100.00	NA
	Saprobity^X^	**0.0747**	5	219	96.57	2.73
	Substrate	**0.0745**	9	224	86.42	8.05
	Temperature	**0.0715**	3	221	85.93	14.07
	Transversal distribution^X^	**0.0941**	6	224	78.74	19.58
	Trophic status	0.0200	3	223	96.86	3.14
	Velocity	**0.1024**	4	224	96.53	3.33

*Note*: The RV‐coefficient indicates the level of co‐structure between the two sets of traits, with bold lettering indicating a significance at α = 0.05 (*p*‐values were adjusted for multiple testing after Bonferroni correction). *n*_mod_ indicates the number of trait modalities, *n*_taxa_ indicates the number of taxa included in the corresponding analysis. Additionally, the proportion of projected inertia on the first two axes is given. Traits marked with an exposed “x” correspond to traits for which we had no clear a priori hypotheses or expected a strong effect of phylogeny and whose detailed results can be found in the Supplementary Material.

### 3.2 Development‐related traits

#### 3.2.1 Life duration

**FIGURE 1:**
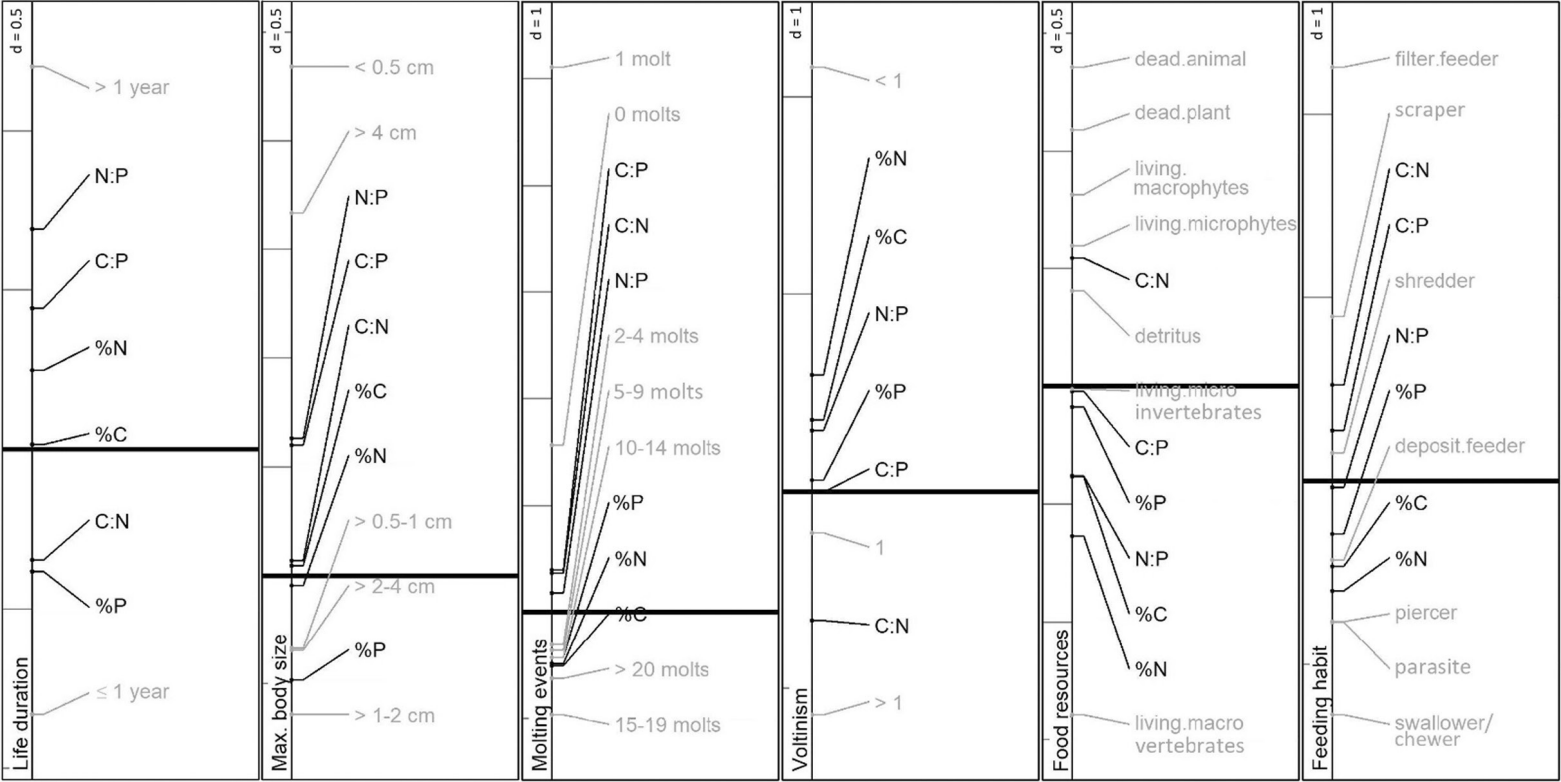
Results from co‐inertia analyses on single biological traits. For each trait, the trait categories (gray) and stoichiometric traits (black) are positioned along the first co‐inertia axis, which captured most of the inertia. The thick horizontal line marks zero; gray lines at the left of each panel help to estimate the strength of the associations with the value for “*d*” indicating the distance between lines.

Having a life duration of ≤1 year was associated with high %P and C:N, all being negatively related to the first axis (Figure 1). A life duration of >1 year was positively related to the first axis as were %N, C:P, and N:P.

#### 3.2.2 Maximum body size

%P was negatively associated with the first axis and thus positively related to small and intermediate maximum sizes (0.5–1, 1–2, 2–4 cm; Figure 1). C:P and N:P were positively related to the first axis, as were taxa of the two extreme categories with the largest (>4 cm) and smallest (<0.5 cm) maximum sizes. %C, %N, and C:N were poorly associated with the first axis.

#### 3.2.3 Molting events

The trait categories of no molting and one molting event were positively associated with the first axis, as were high stoichiometric ratios (C:N, C:P, N:P) (Figure 1). Taxa expressing a high number of molting events (15–19, >20) were strongly and negatively associated with the first axis. Such a negative association was less strong for intermediate numbers of molting events (2–4, 5–9, and 10–14) and high mass element contents.

#### 3.2.4 Voltinism

C:N ratio was the only stoichiometric trait negatively associated with the first axis and was thus co‐varying with taxa exhibiting fast development (>1 generation per year) and to a lesser extent with monovoltin taxa (1 generation per year; Figure 1). %N, %C, and N:P showed the strongest positive associations to the first axis, co‐varying with slow‐developing taxa (<1 generation per year).

### 3.3 Feeding‐traits

#### 3.3.1 Feeding habits

Filter‐feeders exhibited the strongest response and were—together with scrapers and shredders—positively related to the first axis (70.81% of total inertia) and co‐varying with C:N and C:P (Figure 1). Swallowers/chewers, piercers, and parasites were strongly varying in the opposite direction, as were %N, %C, and %P, the latter with a weaker strength (F1 < 0). Deposit‐feeders and shredders were positively varying along the second axis (26.66%), therewith also associated with %P (S4). Taxa with high C:P and N:P were positioned opposite to %P along the second axis (F2 < 0), and thus co‐varied with piercers and parasites.

#### 3.3.2 Food resources

The first axis (79.82% of total inertia) mainly separated %C, %N, and N:P (F1 < 0) from C:N (F1 > 0), the second axis %P (F2 < 0) from C:P and N:P ratio (F2 > 0), still projecting 19.20% of the total inertia (Figure 1; S4). Considering both axes, C:N was associated with taxa feeding on dead animal, while %C, %N, and N:P were strongly and positively co‐varying with taxa using living macroinvertebrates as resources. Taxa feeding on living microinvertebrates and microphytes were strongly related to the second axis (F2 > 0), opposing %P. The trait categories “living macrophytes,” “dead plant,” and “detritus” were slightly varying in this direction (F2 < 0), thus positioned between taxa with high C:N or high %P.

### 3.4 Ecological traits

#### 3.4.1 Altitude

**FIGURE 2:**
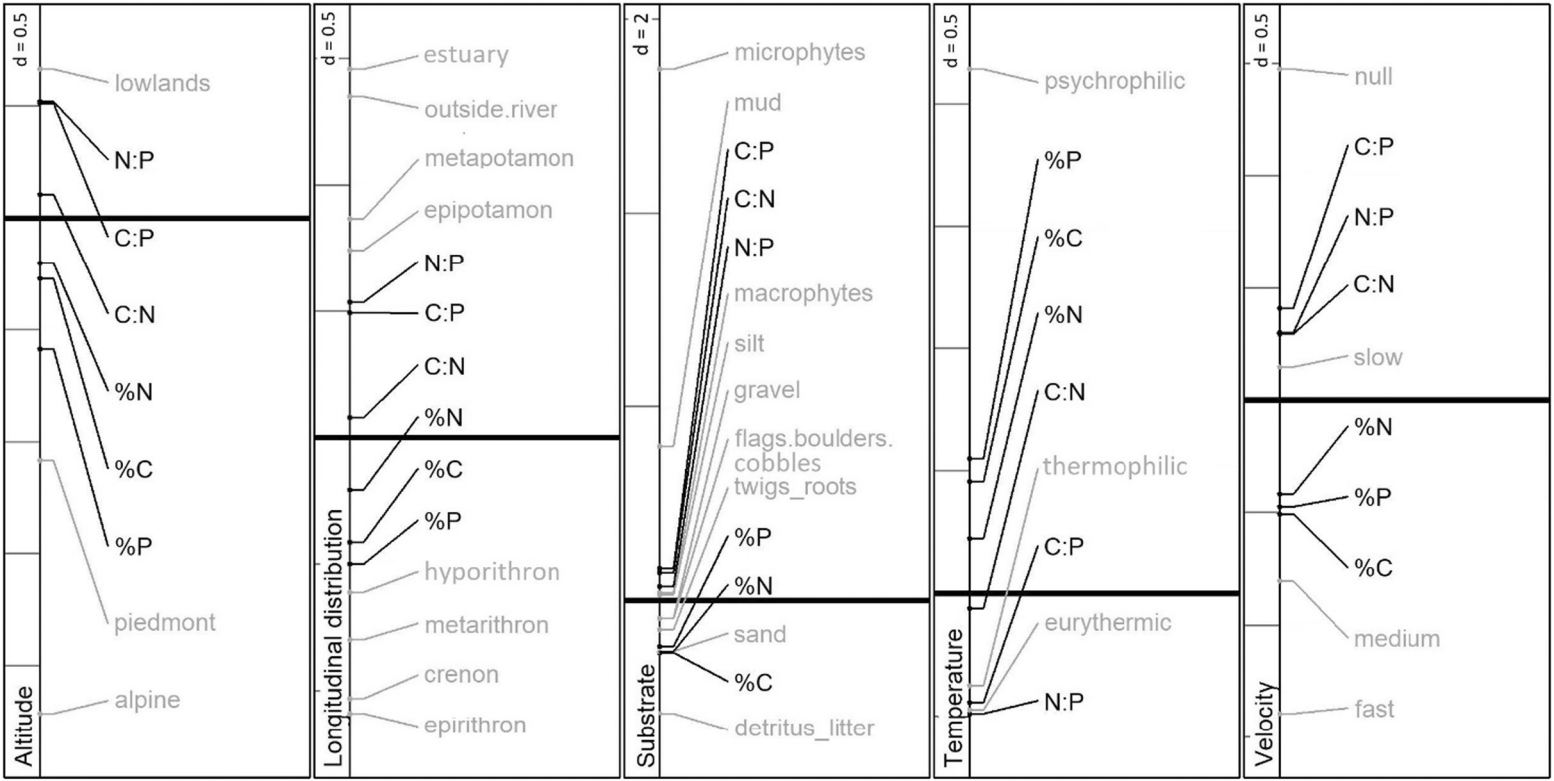
Results from co‐inertia analyses on single ecological traits. For each trait, the trait categories (gray) and stoichiometric traits (black) are positioned along the first co‐inertia axis, which captured most of the inertia. The thick horizontal line marks zero; gray lines at the left of each panel help to estimate the strength of the associations with the value for “*d*” indicating the distance between lines.

Stoichiometric molar ratios and a preference for lowland habitats (F1 > 0) were clearly separated from the mass element contents and preferences for alpine or piedmont habitats (F1 < 0; Figure 2). Among the stoichiometric traits, those related to phosphorus thereby showed the strongest associations.

#### 3.4.2 Longitudinal distribution

%P and %C both varied with the headwater habitats from springs (crenon) to hyporithron along the first axis (F1 < 0; 66.15% of total inertia; Figure 2). Taxa with high C:P and N:P were associated with water bodies disconnected from the river (e.g., ponds, peat bogs, temporary waters) and the potamal (i.e., epi‐ and metapotamon, estuary; F1 > 0). The second axis still explained a large part of co‐inertia (32.26%) and mainly separated %C and %N (F2 < 0) from C:N (F2 > 0). The latter was related to the lower sections of streams (epipotamon and metapotamon) and estuarine habitats, while habitats outside of the stream were strongly and negatively associated with the second axis.

#### 3.4.3 Substrate

The substrate categories “microphytes” and “mud” were strongly and positively associated with the first axis and thus were co‐varying with high C:P, C:N, and N:P ratios (Figure 2). In the other direction, a preference for “detritus/litter” and “sand” was co‐varying with high contents of carbon, nitrogen, and phosphorus.

#### 3.4.4 Temperature

With negative coordinates along the first axis, eurythermic and thermophilic taxa were associated with high C:P and N:P values (Figure 2). Psychrophilic taxa and those rich in %P, %C, and %N were positively associated along the first axis.

#### 3.4.5 Velocity

Taxa preferring standing waters (category “null”) were also taxa exhibiting high stoichiometric ratios (Figure 2). Taxa with high %C, %P, and %N preferred medium to fast current velocities.

Figures related to biological traits without a significant relationship with stoichiometric traits (Table 3) or for which no a priori hypotheses were made (Table 1) can be found in the Supplementary Material (S3).

### 3.5 Complete trait profile

The complete functional trait profiles were significantly related to the stoichiometric trait profiles (RV = 0.27). This relationship was stronger than those observed for single traits and the associations among functional and between functional and stoichiometric traits were—in general—similar to those in single trait analyses described above (S5). The largest part of the co‐structure was explained by the first axis (71.95%), but still 24.58% fell onto the second axis (Table 4). As in (most) single trait analyses variation in stoichiometric profiles was better captured by co‐inertia axes than that of functional traits, whose fit was still >75%.

**TABLE 4:** Results of the co‐inertia analysis between macroinvertebrate complete functional and stoichiometric trait profiles

	RV	P	*n*_traits_	*n*_taxa_	Axis	Projected inertia (%)	Ratio co‐inertia/inertia	
							Fun. traits	Stoichio. traits
Complete	0.2744	0.0017	23	131	Ax 1	71.95	0.7614	0.9787
trait profiles					Ax 2	24.58	0.7731	0.9862
					Ax 3	2.65		

*Note*: The RV‐coefficient indicates the level of co‐structure between the two sets of traits and was significant at α = 0.05. For the first three axes, projected inertia (%) and the ratio between inertia and co‐inertia indicate the fitting level of the analysis. *n*_traits_ and *n*_taxa_ indicate the numbers of functional traits and taxa included in the analysis.

## 4 DISCUSSION

We found significant co‐structures between functional and stoichiometric traits in macroinvertebrates, both when considering macroinvertebrates' complete trait profiles and when analyzing traits individually. Many of these patterns followed our assumptions based on concepts within the ecological stoichiometry framework.

### 4.1 Development‐related traits

We expected that traits corresponding to fast growth or development would be associated with high body phosphorus content. For some trait categories, we were able to show this link: The highest numbers of molting events were positively related to a high phosphorus (and carbon) content. Villar‐Argaiz et al. (2020) found an increasing %P over ontogeny in several hemimetabolous insects and suggested a high phosphorus demand during molts as one explanation. A high number of molting events might also reflect a high growth rate, which, according to GRH, requires high amounts of phosphorus and leads to a high body %P. One example is Ephemeroptera (notably *Baetis*, *Caenis*), which showed high affinities for these trait modalities. Within this insect order, a high number of species live less than 1 year and thus grow and develop rather fast. Additionally, phosphorus content was positively related to small and intermediate maximum body sizes (0.5–1, 1–2 cm) and negatively related to the category of largest maximum sizes (> 4 cm), supporting GRH that small taxa tend to be short‐lived and fast‐growing. However, the category of the smallest maximum body size (<0.5 cm) was associated with low phosphorus content, which was likely mainly driven by small‐sized Coleoptera as they have one of the lowest phosphorus content among insect orders (Beck et al., 2022; Frost et al., 2003; Liess & Hillebrand, 2005). In a field study, Villar‐Argaiz et al. (2020) did not find a correlation between %P and the individual maximum biomass they found for a given taxon. These different results might simply be due to differences in study design (field study with actual measurements vs. a more theoretical approach in our case) and the lower number of taxa included in their study.

When analyzing multi‐trait profiles, slow development, expressed by semivoltinism, long‐life duration (>1 year), and a large number of reproductive events per individual (>6), was related to taxa richer in nitrogen and carbon than slow‐developing ones (e.g., multivoltine). This was also generally observed in the single trait analysis of voltinism where stoichiometric traits were not so well separated, probably because 89% of the taxa can express one generation per year but still differ in their growth rate (slower or faster development).

Some trait categories and their link to body stoichiometry are strongly coupled to phylogeny. Growing without molting (i.e., direct development) or with only one molting event (from planktonic larval to benthic adult instar) was associated with a high C:N ratio. Both are characteristics of Bivalvia and Gastropoda, which generally have a significantly lower nitrogen (and phosphorus) content than other macroinvertebrate groups (Beck et al., 2022; Evans‐White et al., 2005). The association of high C:nutrient ratios with undergoing a complete aquatic life cycle (i.e., aquatic “adults”) and reproducing ovoviviparous or via “fixed cemented clutches” was likely driven by these two groups, even though organisms from other taxonomic groups can also exhibit some of these features.

### 4.2 Feeding‐related traits

Our observations regarding the traits that are linked to food utilization are in line with other studies reporting higher nitrogen content of predators compared with herbivores and detritivores (Cross et al., 2003; Evans‐White et al., 2005; Fagan et al., 2002). Although our study does not use these three categories, we found that swallowers/chewers, preferably feeding on living macroinvertebrates and vertebrates, to which the common term of “predator” applies, were linked to high nitrogen and carbon contents. The reasons are probably the consumption of animal resources, which are more nutrient‐rich (%N, %P) than primary resources and detritus (Cross et al., 2003), as well as a higher proportion of N‐rich muscle tissues that are necessary to forage prey actively moving themselves and/or often exhibiting a patchy distribution. Many of these predators are crawlers, which—although the association was not significant in our study—tended to be related to high nitrogen (and phosphorus) content.

Interestingly, feeding on living macroinvertebrates was not explicitly associated with being rich in phosphorus, even if we had assumed a higher content of phosphorus in this food resource. While Cross et al. (2003) reported this finding, Evans‐White et al. (2005) have not confirmed this observation, similarly to this study. Although several taxa exclusively feed on macroinvertebrates, (e.g., almost all analyzed taxa of Hirudinea and Turbellaria, but also Odonata), other taxa, such as Hydrophilidae (Coleoptera) or Perlodidae (Plecoptera), exhibit a more opportunistic diet (Tachet et al., 2010). Their food spectrum is wider and contains animal prey among other food resources, which can explain the deviation from our expectation.

Scraping the biofilm on surfaces and filter‐feeding were associated with low concentrations of nitrogen (high C:N, opposed to high %N and %C) and are probably influenced by taxonomy, as they are the primary feeding modes of Gastropoda and Bivalvia, respectively, which are generally poor in nitrogen and phosphorus as stated above. Food items that tend to be consumed by nutrient‐poor taxa (e.g., high C:N) included living microphytes, and both animal and plant detritus, that all fall in the potential resource spectrum of these feeding habits. Mehler et al. (2013) reported significantly lower C:N and higher nitrogen content in scrapers compared with gatherers and/or filter‐feeders, but their study only included insects, which probably explains this contrasting finding. Evans‐White et al. (2005) found no difference in stoichiometry among those feeding groups. Our analyses suggest also that they are stoichiometrically similar. Generally, detritus is considered a nutrient‐poor resource compared with other plant or animal‐based food resources and detritivores exhibit low body nutrient concentrations, especially shredders (Cross et al., 2003; Lauridsen et al., 2014).

### 4.3 Ecological traits

For ecological traits, the patterns we found could at least partly be explained by the River Continuum Concept (RCC, Craig, 2002; Vannote et al., 1980)—originally linking invertebrate community composition in terms of functional feeding groups to patterns of geo‐morphological and organic matter properties from river source to mouth—as we hypothesized. This link thereby is indirect via biological traits. For example, organisms' mobility and feeding habits will likely also influence their distribution and substrate preferences.

Taxa with high phosphorus content were associated with preferences for freshwaters, the crenal and metarithral zones, high altitude (>2000 m), and leaf litter or coarse mineral substrates. These are all river characteristics that would occur, according to the RCC, in headwaters. The affinities for large rivers (metapotamon), brackish habitats (estuary), as well as microphytes, mud, and fine mineral substrates—all attributes of downstream river areas—were associated with high C:nutrient ratios (i.e., low nutrient contents %P and %N). The salinity tolerance could also be explained on the cellular level and a lower demand of freshwater organisms for adenosine triphosphate and N‐rich proteins to fuel osmoregulation via the active sodium pump (Lucu & Towle, 2003).

While a priori we had two opposing hypotheses for the link between stoichiometric traits and temperature preference based on metabolic theory—either high nutrient content (%P) due to increased growth rates at higher temperatures (Janssens et al., 2015) or a higher energy demand that results in high C:P and C:N ratios—our results suggest that the latter drove the overall result.

We expected a positive association between %N and %P stoichiometry and eutrophic habitats, due to better nutritional quality—i.e., a higher nutrient content—of basal resources at higher water nutrient concentration (Danger et al., 2016; Demi et al., 2019; Ventura et al., 2008). Nitrogen and phosphorus levels are expected to increase downstream, where the nutrient flow from upstream river reaches and anthropogenic inputs from agriculture are supposed to be higher (Aguilera et al., 2012; Paisley et al., 2011). Our analysis, however, found no significant link but indicates (also in the multi‐trait approach) an association between P‐ and N‐rich taxa and oligotrophic habitats, suggesting that nutrient‐rich taxa are favored in nutrient‐poor rather than nutrient‐rich habitats. Probably those habitats select for taxa that are able to store nutrients in their body tissues, which allows them to survive under rather nutrient‐poor conditions.

Investigating the co‐structure between two sets of traits (here the functional and stoichiometric traits of macroinvertebrates) does not allow to make statements about causation. Some functional traits are clearly depending on taxonomy/phylogeny as already discussed above (e.g., the aquatic life stage(s) and the number of molting events). Thus, trait syndromes related to phylogeny alone could partly explain the observed relationships between functional and stoichiometric traits. However, assumptions from ecological stoichiometry can help explain some of the observed patterns. For example, active, aerial dispersal—only expressed by insects—was linked to high contents of nitrogen, which most likely indicates investment in N‐rich muscle tissues needed for flight. Furthermore, aquatic, passive dispersal, which should require less muscles than active dissemination, was associated with a high C:N (and C:P) ratio and all these traits are—among others and with exceptions—largely prominent in Bivalvia. This clearly demonstrates the difficulty of defining which trait is the “cause” or the “response,” keeping in mind the important role of phylogeny for both types of traits.

### 4.4 Ecological relevance

Understanding the link between functional and stoichiometric traits might help to more clearly understand some mechanisms leading to changes, both in the taxonomic and functional composition of macroinvertebrate assemblages. Considering that shifts in community stoichiometric composition should follow changes in water nutrient concentration (Singer & Battin, 2007; Teurlincx et al., 2017), such changes should go along with shifts in functional trait composition—following the links we have demonstrated. Macroinvertebrates are involved in important functions and ecosystem services at the ecosystem scale (Covich et al., 1999; Wallace & Webster, 1996), contributing to stream secondary production (Poepperl, 2000; Stagliano & Whiles, 2002), organic matter decomposition (Halvorson et al., 2015), or nutrient release via excretion (Knoll et al., 2009). Functional changes could thus lead to changes in nutrient cycling, for example via shorter food chains due to larger body sizes and increased growth rates (Singer & Battin, 2007). Additional field studies along a gradient of nutrient enrichment or the analysis of spatially and/or temporally large‐scale datasets should help to verify that the relationships we found stand for practical application in functional ecology.

While the use of stoichiometric traits in the context of nutrient changes seems rather intuitive due to the direct link between organisms/environment via the elements, it has also the potential to significantly improve the functional diagnostic of biotic communities subjected to other stressors. Temperature increase, deriving from global warming, also favors certain traits, notably small body sizes (Latli et al., 2017; Yvon‐Durocher et al., 2017), and therewith could affect the ecosystem nutrient cycling in a similar way as outlined above. Further, warming increases respiration rates (Hamburger & Dall, 1990) and reduces the nutritional value of autotrophic resources (Domis et al., 2014; Yvon‐Durocher et al., 2017), which could favor taxa with high body C:P and C:N. Knowing associations of stoichiometric traits with (other) functional traits could thus help to understand—or predict—ecosystem functional changes. We think that such an approach could be extended to other ecosystems (e.g., marine, lake, soil) and to other groups of organisms and should improve functional analyses, especially since the stoichiometric approach is already widely used.

Compared with most studies cited in this work, our analysis has covered a greater number of taxa and traits, providing large‐scale results from the macroinvertebrate compartment. These results supported most of our hypotheses based on previous works in EST that (often) did not directly link stoichiometric traits to biological and ecological traits or did so only for a limited number of taxa.

The fact that the single‐ and multi‐trait profile analyses generally revealed the same patterns strengthens our findings. In living organisms, traits appear not independently, and therefore, in complex ecological contexts, the multi‐trait approach is more and more applied (Rodríguez‐Romero et al., 2021; Shah et al., 2022; Wilkes et al., 2020). Similarly, the use of potential, fuzzy coded traits—rather than allocating only one single category to a taxon for a given trait (i.e., a completely disjunctive coding system, e.g., herbivore vs. predator)—has the advantage of more precisely integrating the full intra‐taxon variability that can occur in nature. Many macroinvertebrates can be ecologically flexible and, for example, more or less change their diet according to resource availability (Tomanova et al., 2006; Wellard Kelly et al., 2013; Zhang et al., 2018). Moreover, some of their life‐history characteristics such as their annual number of generations (voltinism) or their number of successive reproductive cycles per individual can vary among populations differently influenced by external factors such as temperature (Altermatt, 2010; Corbet et al., 2006; Lehmkuhl, 2011; Winder et al., 2009).

## 5 CONCLUSION

We have shown that there is a significant co‐occurrence between stoichiometric and biological as well as ecological traits in benthic macroinvertebrates, both when analyzing single traits or overall trait profiles. The associations of traits related to organism development, nutrient cycling (via feeding), or locomotion thereby are in line with assumptions based on concepts and observations within ecological stoichiometry. For ecological traits, the link towards stoichiometric traits is mainly indirect via biological traits such as feeding behavior, additionally displaying the longitudinal stream gradient. Some functional traits have shown a strong effect of phylogeny, which can be explained by group‐specific trait syndromes but partly also by EST.

Demonstrating a common link between stoichiometric and functional traits underlines the potential of integrating stoichiometry into ecological studies to better understand and predict the taxonomic and functional responses of macroinvertebrate communities to environmental changes.

## AUTHOR CONTRIBUTIONS

**Miriam Beck:** Conceptualization (equal); data curation (equal); formal analysis (lead); investigation (equal); writing – original draft (equal); writing – review and editing (equal). **Elise Billoir:** Conceptualization (equal); formal analysis (equal); investigation (equal); writing – original draft (equal); writing – review and editing (equal). **Vincent Felten:** Data curation (equal); investigation (equal); writing – original draft (equal); writing – review and editing (equal). **Albin Meyer:** Data curation (equal); investigation (equal); writing – original draft (equal); writing – review and editing (equal). **Philippe Usseglio‐Polatera:** Conceptualization (equal); data curation (equal); investigation (equal); writing – original draft (equal); writing – review and editing (equal). **Michael Danger:** Conceptualization (equal); formal analysis (equal); funding acquisition (equal); investigation (equal); writing – original draft (equal); writing – review and editing (equal).

## FUNDING INFORMATION

This research was funded by the StoichioMic ANR project (ANR 18 CE32 0003 01) and IUF to MD.

## Supporting information


Appendix S1

## Data Availability

The databases used in this study are freely available, and modifications made are described in the manuscript. The final dataset used in this manuscript is available in Dryad (doi: (added when manuscript is accepted)).
