# Supplementary material for: Lessons from linking bio‐ and ecological traits to stoichiometric traits in stream macroinvertebrates
Source: Ecol Evol. 2022 Dec 8;12(12):e9605. doi: 10.1002/ece3.9605 (PMC9731919; doi:10.1002/ece3.9605)
Supplement: Supplementary file 1 — Appendix S1 [file ECE3-12-e9605-s001.docx]

Supplementary material

S1.: Modifications of trait-categories compared to the original database from Tachet *et al.* (2010).

| Functional Trait | Modification |
| --- | --- |
| Maximum body size | "<0.25 cm" + "0.25-0.5 cm" ⭢ "<0.5 cm" |
|  | "4-8cm" + ">8 cm" ⭢ ">4 cm" |
| Food resources | "living macroinvertebrates" + "vertebrates" ⭢ "(in)vertebrates" |
|  | "detritus" + "microorganisms" ⭢ "detritus" |
| Resistance forms | "housing" + "cocoons" ⭢ "housing/cocoons" |
| Respiration | "plastron" + "spiracle" ⭢ "plastron/spiracle" |
| Locomotion | "surface swimmer" + "full water swimmer" ⭢"swimmer" |
|  | "temporarily attached" + "permanently attached" ⭢ "attached" |
| Transversal distribution | "groundwater" has been removed and relative frequencies calculated without considering this trait category |

S2: List of taxa included in the analysis.

| Taxon | Class | Order | Taxon | Class | Order |
| --- | --- | --- | --- | --- | --- |
| Hydracarina | Arachnida | Hydracarina | Nepidae | Insecta | Hemiptera |
| Arachnida | Arachnida | NA | *Ranatra* | Insecta | Hemiptera |
| *Corbicula* | Bivalvia | NA | Ranatra linearis | Insecta | Hemiptera |
| Corbiculidae | Bivalvia | NA | *Velia* | Insecta | Hemiptera |
| *Dreissena* | Bivalvia | NA | Veliidae | Insecta | Hemiptera |
| Dreissenidae | Bivalvia | NA | Agriotypidae | Insecta | Hymenoptera |
| *Pisidium* | Bivalvia | NA | Sialidae | Insecta | Megaloptera |
| Sphaeriidae | Bivalvia | NA | *Sialis* | Insecta | Megaloptera |
| Ancylidae | Gastropoda | NA | *Aeshna* | Insecta | Odonata |
| *Ancylus* | Gastropoda | NA | Aeshnidae | Insecta | Odonata |
| Ancylus fluviatilis | Gastropoda | NA | *Boyeria* | Insecta | Odonata |
| *Bathyomphalus* | Gastropoda | NA | Calopterygidae | Insecta | Odonata |
| Bathyomphalus contortus | Gastropoda | NA | *Calopteryx* | Insecta | Odonata |
| *Bithynia* | Gastropoda | NA | *Coenagrion* | Insecta | Odonata |
| Bithyniidae | Gastropoda | NA | Coenagrionidae | Insecta | Odonata |
| *Galba* | Gastropoda | NA | Cordulegaster | Insecta | Odonata |
| Galba truncatula | Gastropoda | NA | Cordulegaster boltoni | Insecta | Odonata |
| Lymnaeidae | Gastropoda | NA | Cordulegasteridae | Insecta | Odonata |
| *Menetus* | Gastropoda | NA | Gomphidae | Insecta | Odonata |
| Menetus dilatatus | Gastropoda | NA | *Gomphus* | Insecta | Odonata |
| *Physa* | Gastropoda | NA | *Libellula* | Insecta | Odonata |
| Physidae | Gastropoda | NA | Libellulidae | Insecta | Odonata |
| Planorbidae | Gastropoda | NA | Libellulinae | Insecta | Odonata |
| *Radix* | Gastropoda | NA | *Onychogomphus* | Insecta | Odonata |
| *Stagnicola* | Gastropoda | NA | Platycnemididae | Insecta | Odonata |
| Hirudidae | Hirudinea | Gnathobdelliformes | *Platycnemis* | Insecta | Odonata |
| *Hirudo* | Hirudinea | Gnathobdelliformes | *Amphinemura* | Insecta | Plecoptera |
| Hirudo medicinalis | Hirudinea | Gnathobdelliformes | *Brachyptera* | Insecta | Plecoptera |
| *Erpobdella* | Hirudinea | Pharyngobdelliformes | *Capnia* | Insecta | Plecoptera |
| Erpobdellidae | Hirudinea | Pharyngobdelliformes | Capniidae | Insecta | Plecoptera |
| *Trocheta* | Hirudinea | Pharyngobdelliformes | Chloroperlidae | Insecta | Plecoptera |
| *Glossiphonia* | Hirudinea | Rhynchobdelliformes | *Dinocras* | Insecta | Plecoptera |
| Glossiphoniidae | Hirudinea | Rhynchobdelliformes | Dinocras cephalotes | Insecta | Plecoptera |
| *Helobdella* | Hirudinea | Rhynchobdelliformes | *Isoperla* | Insecta | Plecoptera |
| Helobdella stagnalis | Hirudinea | Rhynchobdelliformes | *Leuctra* | Insecta | Plecoptera |
| *Hemiclepsis* | Hirudinea | Rhynchobdelliformes | Leuctridae | Insecta | Plecoptera |
| Hemiclepsis marginata | Hirudinea | Rhynchobdelliformes | *Nemoura* | Insecta | Plecoptera |
| *Piscicola* | Hirudinea | Rhynchobdelliformes | Nemouridae | Insecta | Plecoptera |
| Piscicola geometra | Hirudinea | Rhynchobdelliformes | *Perla* | Insecta | Plecoptera |
| Piscicolidae | Hirudinea | Rhynchobdelliformes | Perlidae | Insecta | Plecoptera |
| *Agabus* | Insecta | Coleoptera | *Perlodes* | Insecta | Plecoptera |
| *Berosus* | Insecta | Coleoptera | Perlodidae | Insecta | Plecoptera |
| Colymbetinae | Insecta | Coleoptera | *Protonemura* | Insecta | Plecoptera |
| Dytiscidae | Insecta | Coleoptera | Protonemura meyeri | Insecta | Plecoptera |
| Dytiscinae | Insecta | Coleoptera | *Siphonoperla* | Insecta | Plecoptera |
| *Dytiscus* | Insecta | Coleoptera | Taeniopterygidae | Insecta | Plecoptera |
| Elmidae | Insecta | Coleoptera | *Taeniopteryx* | Insecta | Plecoptera |
| *Elmis* | Insecta | Coleoptera | *Adicella* | Insecta | Trichoptera |
| *Elodes* | Insecta | Coleoptera | Agapetinae | Insecta | Trichoptera |
| Haliplidae | Insecta | Coleoptera | *Agapetus* | Insecta | Trichoptera |
| *Haliplus* | Insecta | Coleoptera | *Allogamus* | Insecta | Trichoptera |
| Hydraenidae | Insecta | Coleoptera | *Athripsodes* | Insecta | Trichoptera |
| Hydrophilidae | Insecta | Coleoptera | Brachycentridae | Insecta | Trichoptera |
| Hydrophilinae | Insecta | Coleoptera | *Brachycentrus* | Insecta | Trichoptera |
| Hydroporinae | Insecta | Coleoptera | *Chimarra* | Insecta | Trichoptera |
| Laccophilinae | Insecta | Coleoptera | Chimarrinae | Insecta | Trichoptera |
| *Laccophilus* | Insecta | Coleoptera | *Diplectrona* | Insecta | Trichoptera |
| *Limnius* | Insecta | Coleoptera | Drusinae | Insecta | Trichoptera |
| *Macronychus* | Insecta | Coleoptera | *Drusus* | Insecta | Trichoptera |
| Noteridae | Insecta | Coleoptera | *Glossosoma* | Insecta | Trichoptera |
| *Noterus* | Insecta | Coleoptera | Glossosomatidae | Insecta | Trichoptera |
| *Oulimnius* | Insecta | Coleoptera | *Goera* | Insecta | Trichoptera |
| *Platambus* | Insecta | Coleoptera | Goera pilosa | Insecta | Trichoptera |
| Scirtidae | Insecta | Coleoptera | Goeridae | Insecta | Trichoptera |
| *Stenelmis* | Insecta | Coleoptera | *Hydropsyche* | Insecta | Trichoptera |
| *Antocha* | Insecta | Diptera | Hydropsychidae | Insecta | Trichoptera |
| Athericidae | Insecta | Diptera | Hydroptilidae | Insecta | Trichoptera |
| *Atherix* | Insecta | Diptera | *Lepidostoma* | Insecta | Trichoptera |
| Ceratopogonidae | Insecta | Diptera | Lepidostomatidae | Insecta | Trichoptera |
| Ceratopogoninae | Insecta | Diptera | Leptoceridae | Insecta | Trichoptera |
| Chironomidae | Insecta | Diptera | Limnephilidae | Insecta | Trichoptera |
| Chironominae | Insecta | Diptera | Limnephilinae | Insecta | Trichoptera |
| Chironomini | Insecta | Diptera | Limnephilini | Insecta | Trichoptera |
| *Chironomus* | Insecta | Diptera | *Limnephilus* | Insecta | Trichoptera |
| Chironomus plumosus | Insecta | Diptera | *Mesophylax* | Insecta | Trichoptera |
| *Dicranota* | Insecta | Diptera | *Mystacides* | Insecta | Trichoptera |
| *Dixa* | Insecta | Diptera | *Nemotaulius* | Insecta | Trichoptera |
| Dixidae | Insecta | Diptera | Nemotaulius punctatolineatus | Insecta | Trichoptera |
| Empididae | Insecta | Diptera | Odontoceridae | Insecta | Trichoptera |
| Eriopterini | Insecta | Diptera | *Odontocerum* | Insecta | Trichoptera |
| Hexatoma | Insecta | Diptera | Odontocerum albicorne | Insecta | Trichoptera |
| Hexatomini | Insecta | Diptera | *Oecetis* | Insecta | Trichoptera |
| *Ibisia* | Insecta | Diptera | Philopotamidae | Insecta | Trichoptera |
| Limoniidae | Insecta | Diptera | *Philopotamus* | Insecta | Trichoptera |
| Limoniini | Insecta | Diptera | *Plectrocnemia* | Insecta | Trichoptera |
| Orthocladiinae | Insecta | Diptera | Plectrocnemia conspersa | Insecta | Trichoptera |
| Pediciini | Insecta | Diptera | Polycentropodidae | Insecta | Trichoptera |
| Psychodidae | Insecta | Diptera | Polycentropodinae | Insecta | Trichoptera |
| Ptychopteridae | Insecta | Diptera | *Potamophylax* | Insecta | Trichoptera |
| Simuliidae | Insecta | Diptera | Potamophylax cingulatus | Insecta | Trichoptera |
| Simuliinae | Insecta | Diptera | Psychomyiidae | Insecta | Trichoptera |
| Simuliini | Insecta | Diptera | *Ptilocolepus* | Insecta | Trichoptera |
| Stratiomyidae | Insecta | Diptera | Ptilocolepus granulatus | Insecta | Trichoptera |
| Tabanidae | Insecta | Diptera | *Rhyacophila* | Insecta | Trichoptera |
| *Tipula* | Insecta | Diptera | Rhyacophilidae | Insecta | Trichoptera |
| Tipula lateralis | Insecta | Diptera | *Sericostoma* | Insecta | Trichoptera |
| Tipulidae | Insecta | Diptera | Sericostomatidae | Insecta | Trichoptera |
| Ameletidae | Insecta | Ephemeroptera | Silo-Lithax | Insecta | Trichoptera |
| *Ameletus* | Insecta | Ephemeroptera | Stenophylacini-Chaetopterygini | Insecta | Trichoptera |
| Baetidae | Insecta | Ephemeroptera | *Tinodes* | Insecta | Trichoptera |
| *Baetis* | Insecta | Ephemeroptera | Tinodes waeneri | Insecta | Trichoptera |
| Caenidae | Insecta | Ephemeroptera | Corophiidae | Malacostraca | Amphipoda |
| *Caenis* | Insecta | Ephemeroptera | *Corophium* | Malacostraca | Amphipoda |
| *Ecdyonurus* | Insecta | Ephemeroptera | *Dikerogammarus* | Malacostraca | Amphipoda |
| *Epeorus* | Insecta | Ephemeroptera | *Echinogammarus* | Malacostraca | Amphipoda |
| *Ephemera* | Insecta | Ephemeroptera | Echinogammarus berilloni | Malacostraca | Amphipoda |
| Ephemera danica | Insecta | Ephemeroptera | Gammaridae | Malacostraca | Amphipoda |
| *Ephemerella* | *Insecta* | *Ephemeroptera* | *Gammarus* | *Malacostraca* | *Amphipoda* |
| Ephemerella ignita | Insecta | Ephemeroptera | Gammarus fossarum | Malacostraca | Amphipoda |
| Ephemerella major | Insecta | Ephemeroptera | Gammarus pulex | Malacostraca | Amphipoda |
| Ephemerella mucronata | Insecta | Ephemeroptera | Gammarus roeseli | Malacostraca | Amphipoda |
| Ephemerellidae | Insecta | Ephemeroptera | Pontogammaridae | Malacostraca | Amphipoda |
| Ephemeridae | Insecta | Ephemeroptera | *Atyaephyra* | Malacostraca | Decapoda |
| *Habroleptoides* | Insecta | Ephemeroptera | Atyidae | Malacostraca | Decapoda |
| *Habrophlebia* | Insecta | Ephemeroptera | Cambaridae | Malacostraca | Decapoda |
| *Heptagenia* | Insecta | Ephemeroptera | Orconectes | Malacostraca | Decapoda |
| Heptageniidae | Insecta | Ephemeroptera | Asellidae | Malacostraca | Isopoda |
| *Leptophlebia* | Insecta | Ephemeroptera | *Asellus* | Malacostraca | Isopoda |
| Leptophlebiidae | Insecta | Ephemeroptera | Asellus aquaticus | Malacostraca | Isopoda |
| *Paraleptophlebia* | Insecta | Ephemeroptera | Atyaephyra desmaresti | NA | NA |
| *Rhithrogena* | Insecta | Ephemeroptera | Oligochaeta | Oligochaeta | NA |
| *Serratella* | Insecta | Ephemeroptera | Dendrocoelidae | Turbellaria | Tricladida |
| Siphlonuridae | Insecta | Ephemeroptera | *Dendrocoelum* | Turbellaria | Tricladida |
| *Siphlonurus* | Insecta | Ephemeroptera | Dendrocoelum lacteum | Turbellaria | Tricladida |
| *Torleya* | Insecta | Ephemeroptera | *Dugesia* | Turbellaria | Tricladida |
| Aphelocheiridae | Insecta | Hemiptera | Dugesiidae | Turbellaria | Tricladida |
| *Aphelocheirus* | Insecta | Hemiptera | Planariidae | Turbellaria | Tricladida |
| Aphelocheirus aestivalis | Insecta | Hemiptera | *Polycelis* | Turbellaria | Tricladida |
| *Nepa* | Insecta | Hemiptera |  |  |  |


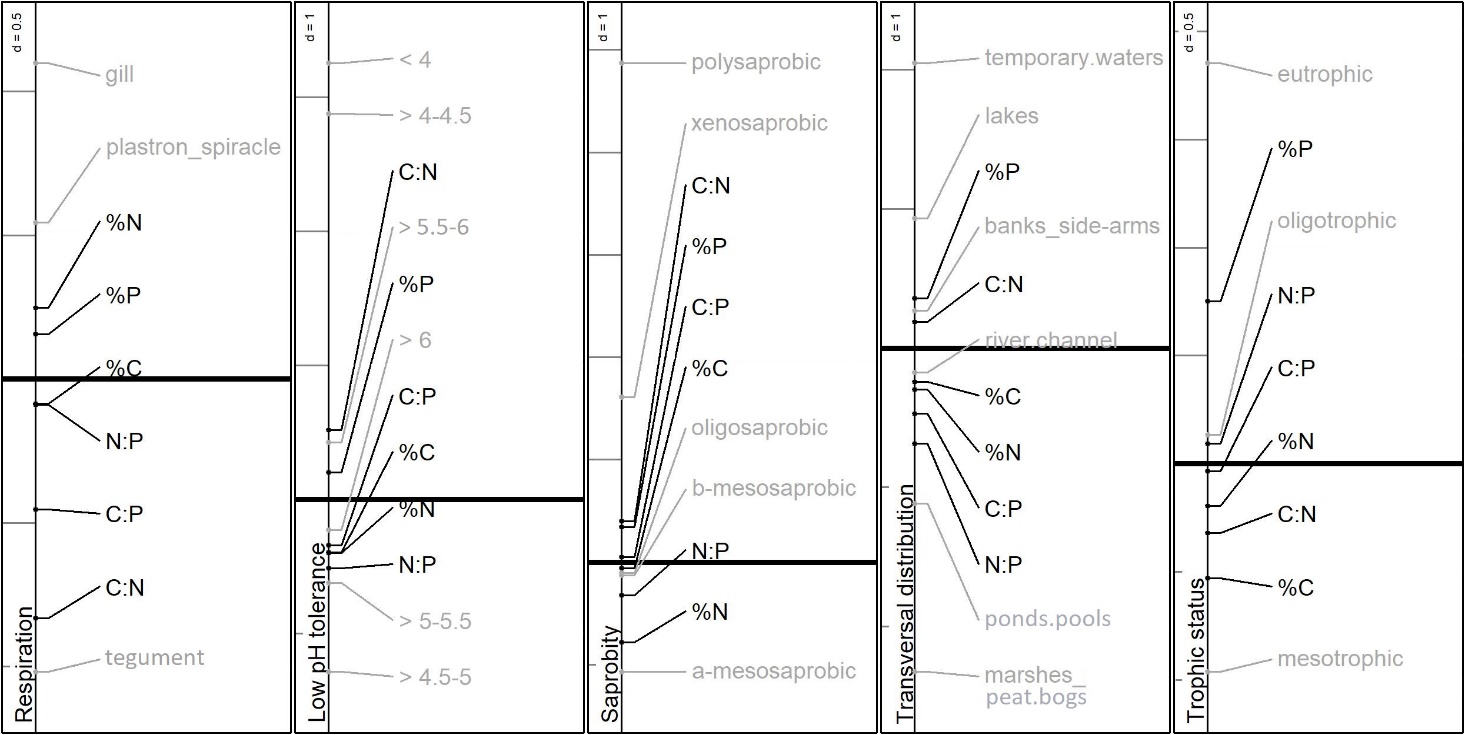

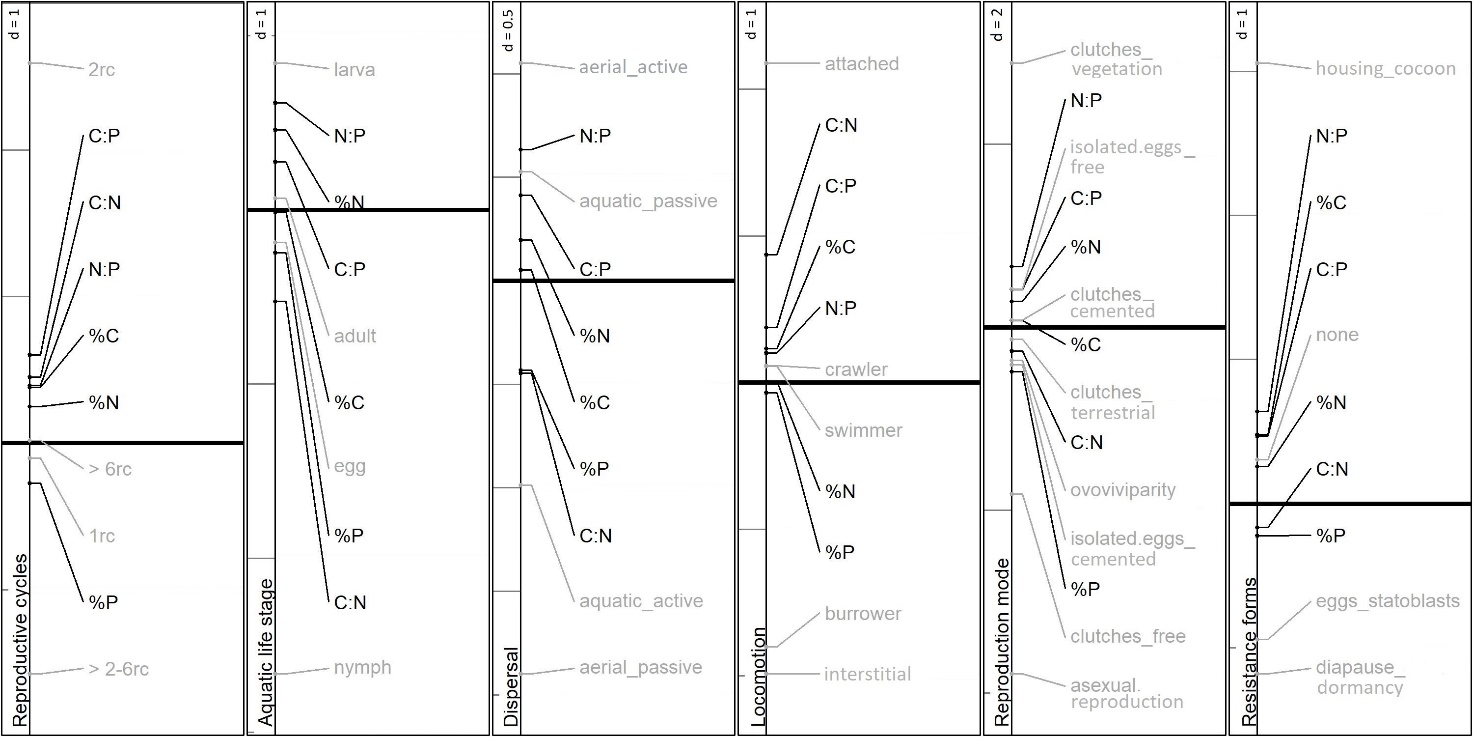
S3: Results from co-inertia analyses on single biological and ecological traits for which we had no *a priori* hypothesis and/or for which the results were not significant (“ns”). For each functional trait, the trait categories (gray) and stoichiometric traits (black) are positioned along the first co-inertia axis, which captured most of the inertia. The thick horizontal line marks zero; gray lines at the left of each panel help to estimate the strength of the associations with the value for “d” indicating the distance between lines.


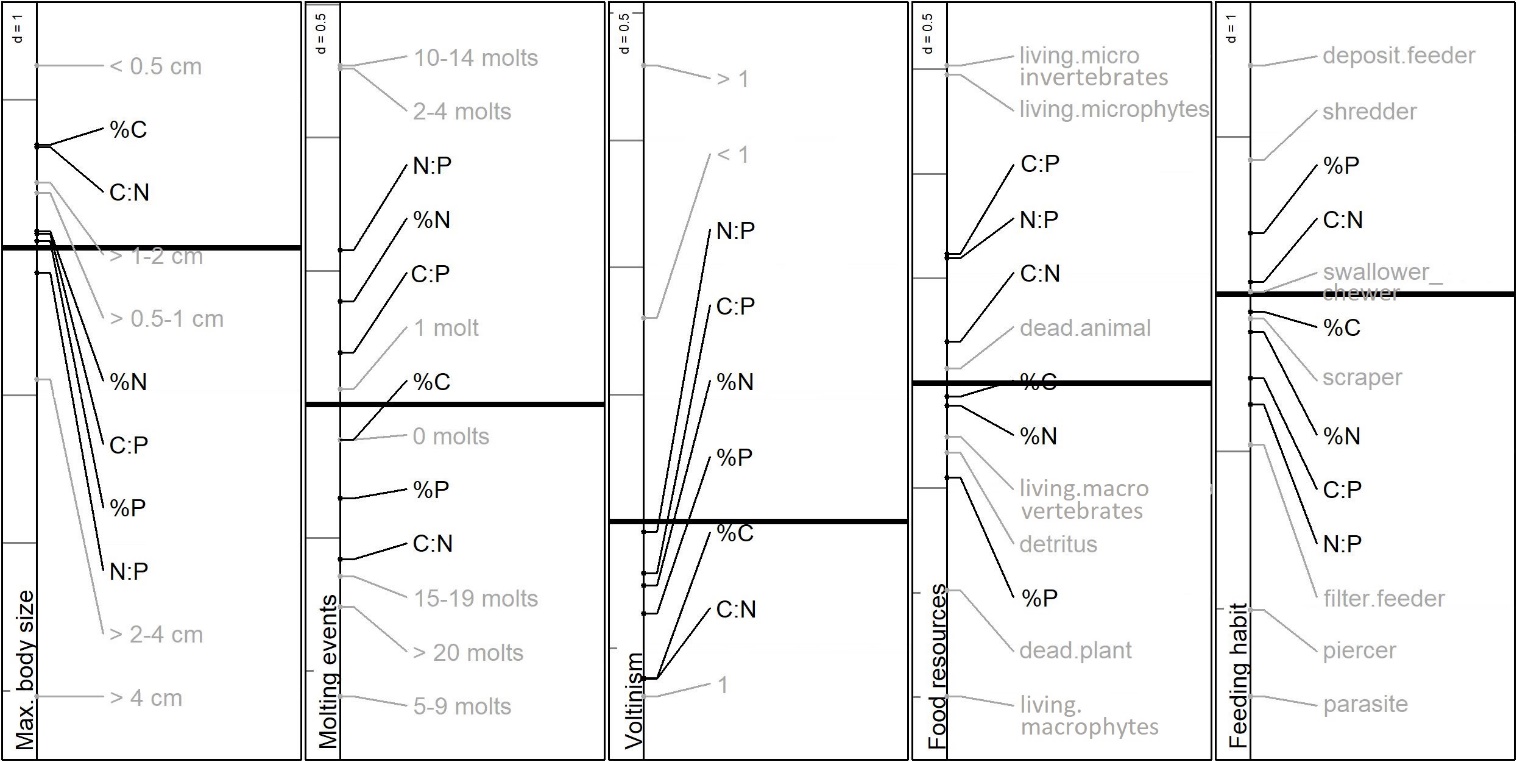

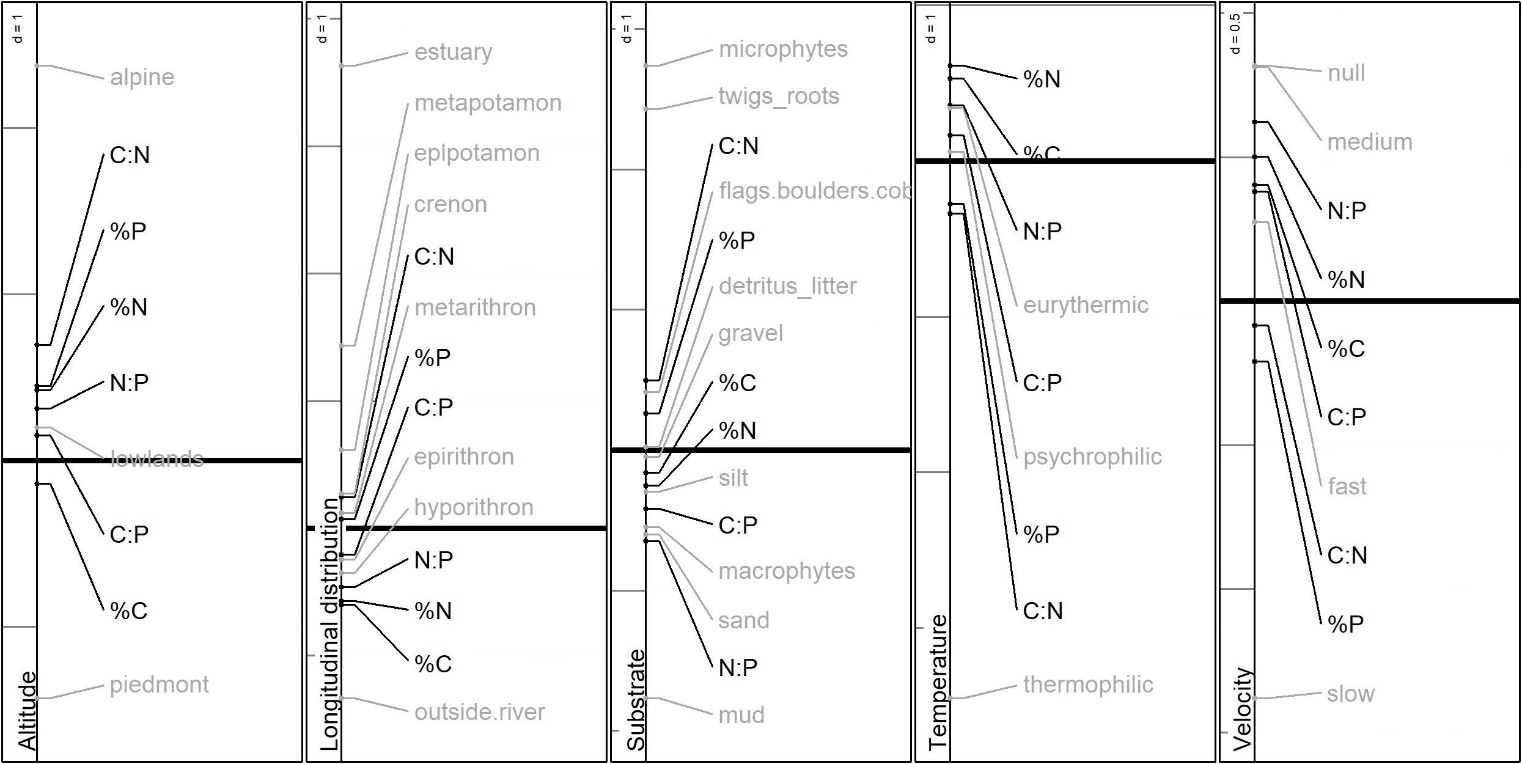


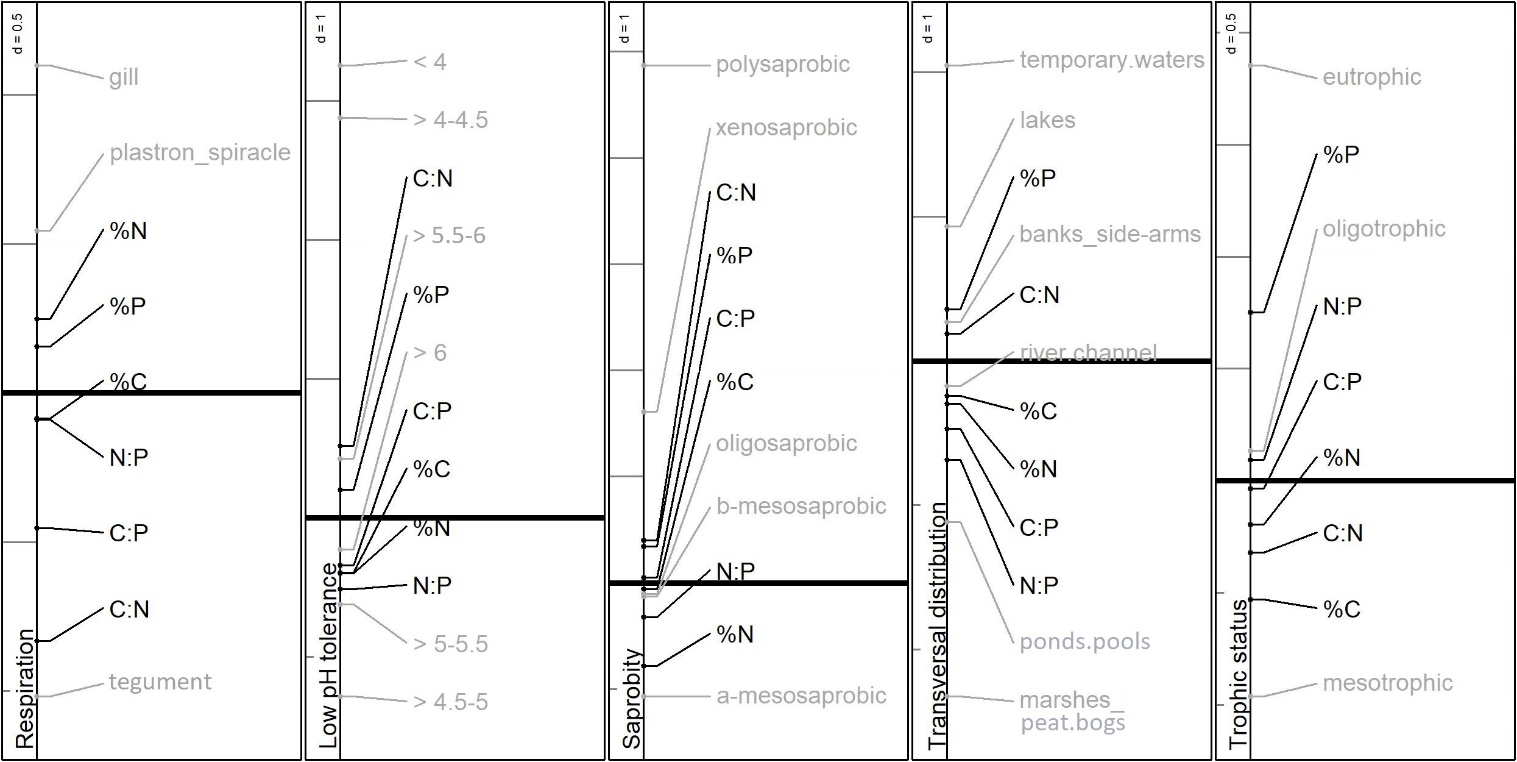

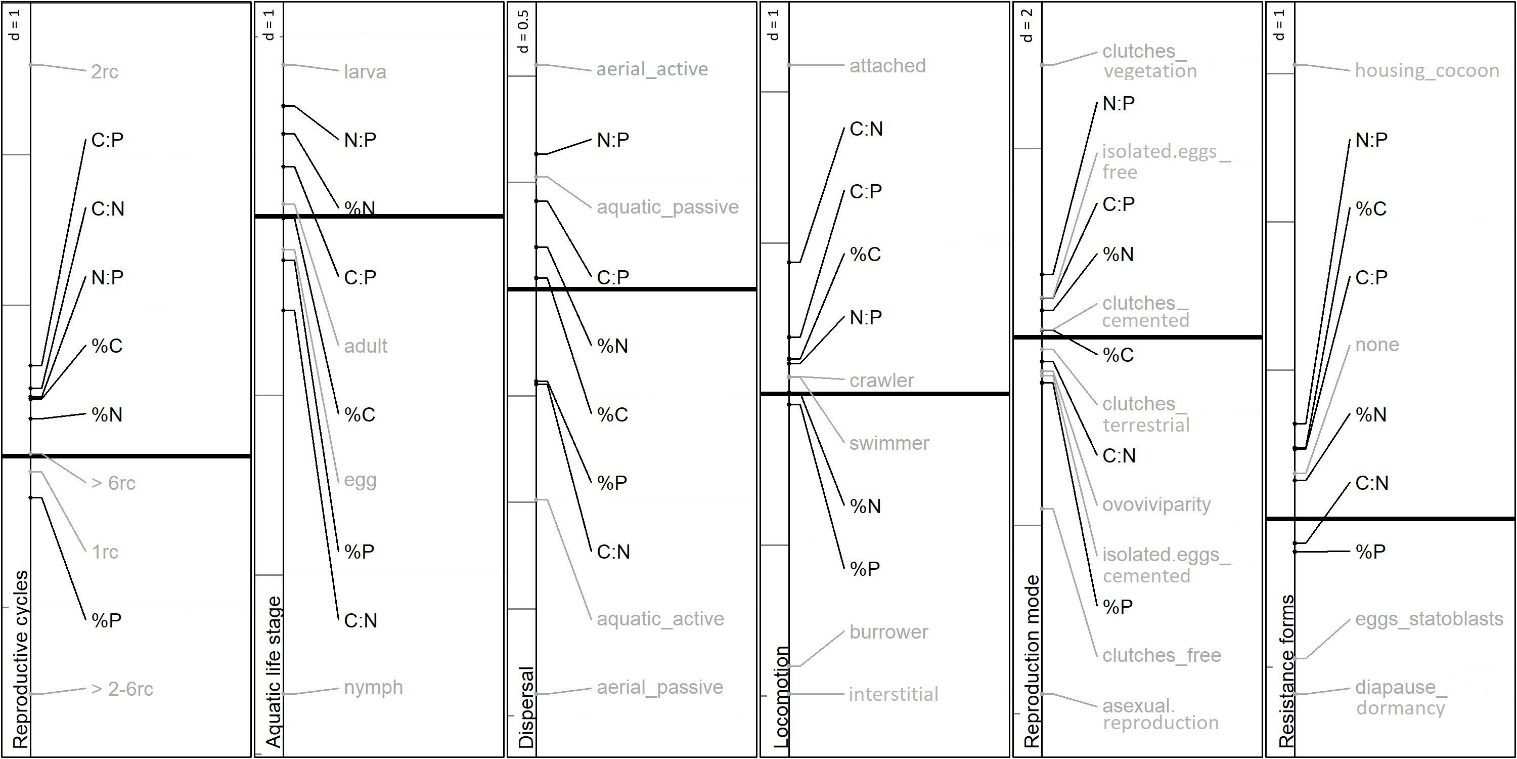
S4: Results from co-inertia analyses on single biological and ecological traits. The upper ten panels show the functional traits also presented in detail in the main manuscript, the lower eleven panels those for which we had no *a priori* hypothesis and/or for which the results were not significant (“ns”). For each functional trait, the trait categories (gray) and stoichiometric traits (black) are positioned along the second co-inertia axis. The thick horizontal line marks zero; gray lines at the left of each panel help to estimate the strength of the associations with the value for “d” indicating the distance between lines. The traits defined by only two categories (life duration, salinity tolerance) are not represented since the total inertia is ultimately captured by one axis only.


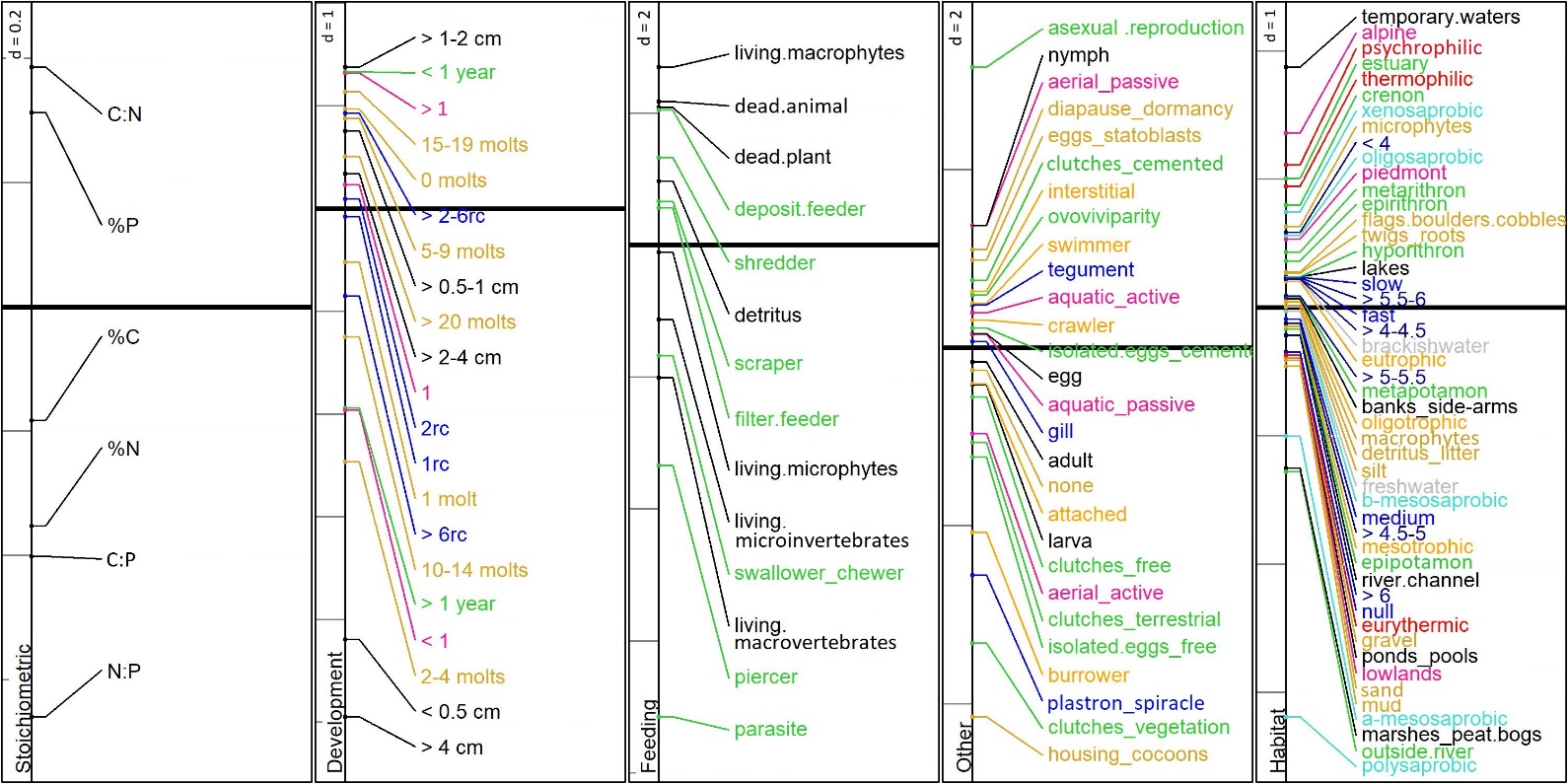

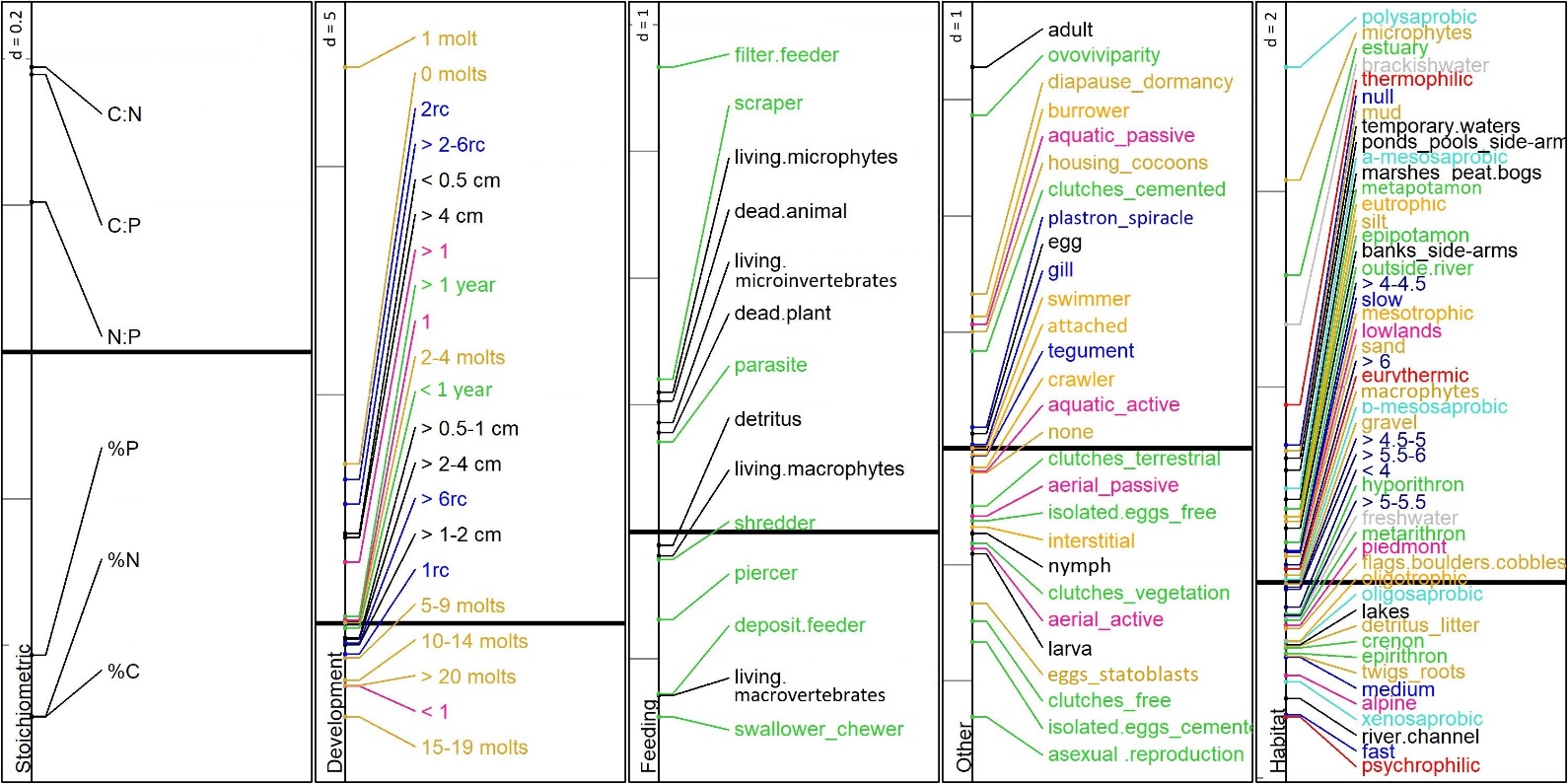
S5: Results from co-inertia analyses on multi-trait profiles comprising all biological and ecological traits. The stoichiometric traits and functional trait categories are positioned along the first (upper panel) or second (lower panel) co-inertia axis. The thick horizontal line in each panel marks zero; gray lines at the left of each panel help to estimate the strength of the associations with the value for “d” indicating the distance between lines. Note that all traits were part of one analysis and simple faceted according to the four trait groups as discussed separately throughout the manuscript (development, feeding, other, habitat) and stoichiometric traits for easier readability.
